# Endogenous Neurosteroid (3α,5α)3-Hydroxypregnan-20-one Inhibits Toll-like-4 Receptor Activation and Pro-inflammatory Signaling in Macrophages and Brain

**DOI:** 10.1038/s41598-018-37409-6

**Published:** 2019-02-04

**Authors:** Irina Balan, Matthew C. Beattie, Todd K. O’Buckley, Laure Aurelian, A. Leslie Morrow

**Affiliations:** 1 Department of Pharmacology, University of Maryland School of Medicine, Baltimore, Maryland USA; 2 Department of Psychiatry and Pharmacology, Bowles Center for Alcohol Studies, University of North Carolina at Chapel Hill, Chapel Hill, North Carolina USA

## Abstract

The endogenous neurosteroid (3α,5α)3-hydroxypregnan-20-one (3α,5α-THP, allopregnanolone) has protective activity in animal models of alcoholism, depression, traumatic brain injury, schizophrenia, multiple sclerosis, and Alzheimer’s disease that is poorly understood. Because these conditions involve proinflammatory signaling through toll-like receptors (TLRs), we examined the effects of 3α,5α-THP, and pregnenolone on TLR4 activation in both the periphery and the central nervous system (CNS). We used monocytes/macrophages (RAW264.7) as a model of peripheral immune signaling and studied innately activated TLR4 in the ventral tegmental area (VTA) of selectively bred alcohol-preferring (P) rats. LPS activated the TLR4 pathway in RAW264.7 cells as evidenced by increased levels of p-TAK1, TRAF6, NF-κB p50, phospho-NF-κB- p65, pCREB, HMGB1, and inflammatory mediators, including MCP-1 and TNFα. Both 3α,5α-THP and pregnenolone (0.5–1.0μM) substantially (~80%) inhibited these effects, indicating pronounced inhibition of TLR4 signaling. The mechanism of inhibition appears to involve blockade of TLR4/MD-2 protein interactions in RAW246.7 cells. In VTA, 3α,5α-THP (15 mg/kg, IP) administration reduced TRAF6 (~20%), CRF (~30%), and MCP-1 (~20%) levels, as well as TLR4 binding to GABA_A_ receptor α2 subunits (~60%) and MyD88 (~40%). The data suggest that inhibition of proinflammatory neuroimmune signaling underlies protective effects of 3α,5α-THP in immune cells and brain, apparently involving blocking of protein-protein interactions that initiate TLR4-dependent signaling. Inhibition of pro-inflammatory TLR4 activation represents a new mechanism of 3α,5α-THP action in the periphery and the brain.

## Introduction

Neurosteroids are endogenous steroids synthesized in brain that influence neuronal and behavioral activity. First recognized by Hans Selye^1^, various neurosteroids were found to alter CNS activity. Later studies showed that endogenous steroids (3α,5α)3-hydroxypregnan-20-one (3α,5α-THP, allopregnanolone) and (3α,5α)3,21-dihydroxypregnan-20-one (3α,5α-THDOC, tetrahydrodeoxycorticosterone), lack genomic activity at nuclear glucocorticoid or progesterone receptors^2^, but are potent positive modulators of GABA_A_ receptors^3,4^. They act upon synaptic and extrasynaptic γ-aminobutyric acid A receptors (GABA_A_Rs), mediating both phasic and tonic inhibition^5,6^. Consistent with their GABAergic activity, these steroids have anesthetic, anticonvulsant, sedative, and anxiolytic effects^7^, and modulate the hypothalamic pituitary adrenal axis to reduce stress activation^8,9^. More recent findings indicate that 3α,5α-THP has beneficial activities in rat and monkey models of alcoholism^10,11^, traumatic brain injury^12^, multiple sclerosis^13,14^, and Alzheimer’s disease^15^. Significantly, pregnenolone, progesterone, and/or 3α,5α-THP also have efficacy in clinical studies of traumatic brain injury^16^, schizophrenia^17^, cocaine craving^18,19^, and post-partum depression^20^. However, the mechanism of these actions is unknown.

Neuroimmune signaling in the brain elevates proinflammatory cytokines, chemokines, and their associated receptors to promote CNS disease in a progressive feed-forward manner^21^. Proinflammatory signaling through toll-like 4 receptors (TLR4) is elevated in physiological stress^22^ and traumatic brain injury^23,24^ and it contributes to the aforementioned neuropsychiatric diseases, including alcohol use disorders^25,26^, other addictions^27^, depression^28,29^, and epilepsy^30^.

In macrophages, the TLR4-specific ligand, lipopolysaccharide (LPS), causes receptor oligomerization at the cell membrane, inducing a cascade of protein-protein interactions that produce proinflammatory cytokines and chemokines. LPS-activation of TLR4 signaling involves formation of a TLR4/MD-2 (myeloid differentiation factor 2) complex that is followed by intracellular signals, including the myeloid differentiation primary response 88 (MyD88)-dependent pathway that activates tumor necrosis factor receptor associated factor 6 (TRAF6), transforming growth factor (TGF)-β-activated kinase 1 (TAK1), and transcription factors NF-κB and cyclic AMP response element binding protein (CREB). Activated transcription factors translocate to the nucleus and initiate a proinflammatory response that involves the production of chemokines and various proinflammatory cytokines^31–35^. Peripheral inflammation also induces pro-inflammatory signaling in the brain^36–39^.

The TLR4 signal is also activated in neurons^40–43^, but the activation mechanism in these cells, the identity of the pathway members, and their similarity to the canonical pathway previously established in immune cells^31–35,44^ are still unclear. The TLR4 signal is innately activated in CNS neurons from male P rats selectively bred for alcohol intake, but not in alcohol-non-preferring (NP) or Wistar rats^41–43,45^. The TLR4 signal involves the GABA_A_R α2 subunit and corticotropin releasing hormone (CRF), known to promote TLR4 signaling^41,46,47^, and it controls impulsivity and the initiation of binge alcohol drinking^41–43^. Both stress and alcohol induce CRF signaling and both play a significant role in addiction^48–52^, as well as other neuropsychiatric diseases.

To examine the possibility that 3α,5α-THP inhibits proinflammatory neuroimmune signaling in the periphery, we studied the effects of 3α,5α-THP and pregnenolone on LPS-induced TLR4 activation and pro-inflammatory signaling in mouse monocyte/macrophage RAW264.7 cells. To avoid potential effects of peripheral immune activation on inflammatory signaling in the brain, we studied TLR4 signals and CRF expression in the VTA of naïve male P rats, which exhibit innate (LPS-independent) TLR4 activation, as described above. We focused on the VTA because both TLR4 and neuroactive steroid modulation in the VTA alter drinking behavior^10,41^. Pregnenolone was tested because it shares the same steroid ring D structure of 3α,5α-THP, but lacks intrinsic potent GABAergic activity^6,53^. 3α,5α-THP also inhibits CRF-mediated activation of the hypothalamic pituitary adrenal axis^9,54^, but effects on extra-hypothalamic CRF are unknown. Finally, we examined the effects of the steroids on the TLR activation mechanisms in both RAW264.7 cells and P rat brain.

## Results

### 3α,5α-THP and pregnenolone inhibit LPS-activated TLR4 signaling in RAW264.7 cells

To examine the neurosteroids’ effect on TLR4 signal activation, RAW264.7 cells were treated with LPS (1 µg/ml; 24 hrs) in the absence or presence of 3α,5α-THP (0.5 µM, 1 µM) or pregnenolone (0.5 µM, 1 µM), and cell extracts were assayed for expression of MyD88-dependent pathway members, by immunoblotting with antibodies to p-TAK1, monocyte chemotactic protein (MCP-1), TRAF6, TLR4, and transcription factor NF-κB p50^31–33^. Cell viability was assessed by the trypan blue exclusion assay, as described in Materials and Methods.

**Figure 1:**
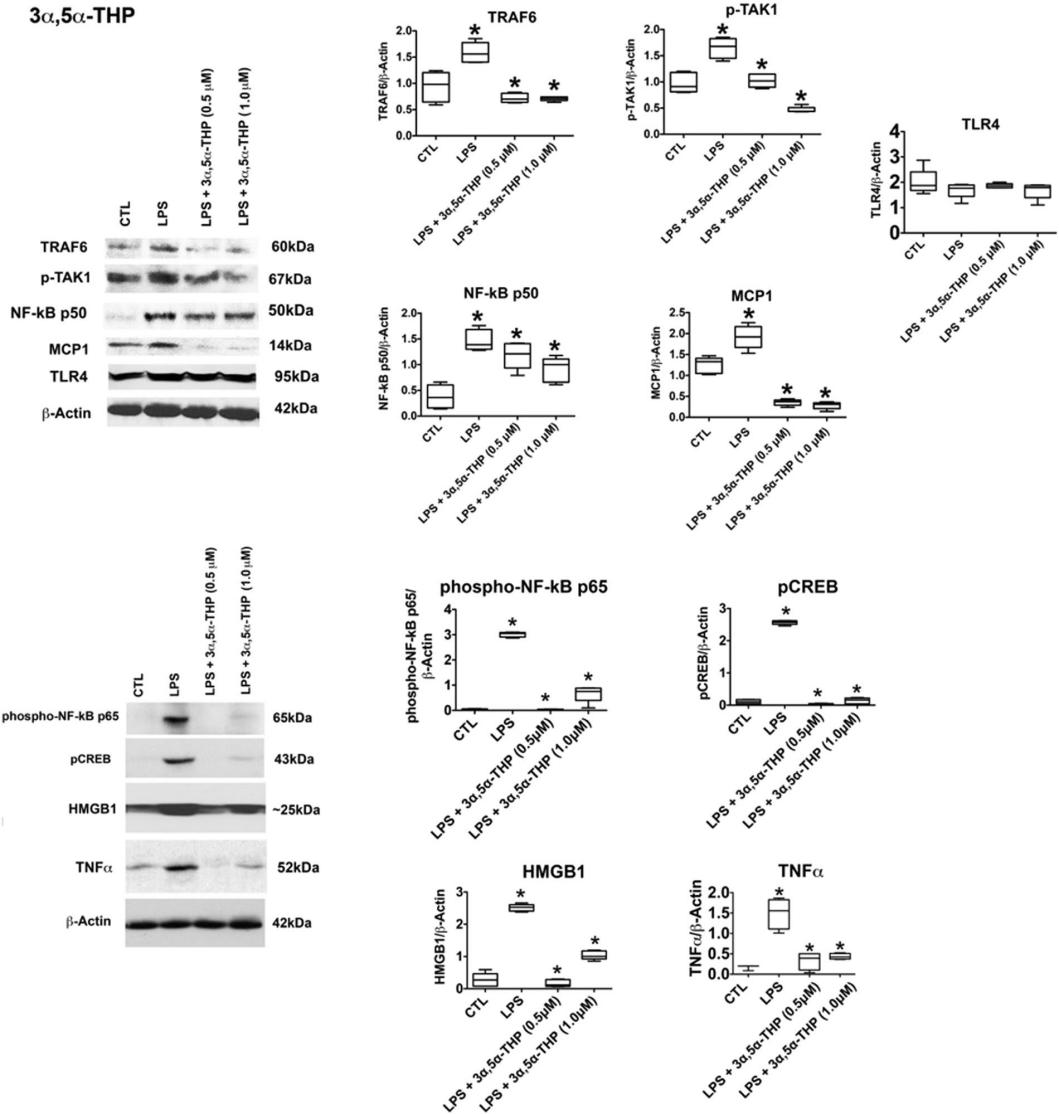
3α,5α-THP inhibits LPS-activated TLR4 signaling in RAW264.7 cells. 3α,5α-THP inhibits LPS-activated TLR4 signaling in RAW264.7 cells. RAW264.7 cells were treated with LPS alone (1 µg/ml) or LPS together with 3α,5α-THP (0.5 µM or 1 µM) and harvested after 24 hrs. The levels of p-TAK1 [F_19_ = 50.47, n = 5/grp], MCP-1 [F_19_ = 97.27, n = 5/grp], TRAF6 [F_19_ = 26.54, n = 5/grp], NF-κB p50 [F_19_ = 19.89, n = 5/grp], phospho-NF-κB p65 [F_19_ = 37.95, n = 5/grp], pCREB [F_19_ = 89.06, n = 5/grp], HMGB1 [F_19_ = 19.64, n = 5/grp], and TNFα [F_15_ = 29.62, n = 4/grp] were significantly increased in LPS-treated vs. untreated cells (CTL), but the increase was inhibited with 3α,5α-THP at both doses studied (*p ≤ 0.05, by One-way ANOVA; Newman-Keuls post-hoc test). 3α,5α-THP (0.5 µM, p = 0.3385, n = 5/grp or 1 µM, p = 0.6947, n = 5/grp) did not affect TLR4 expression. Blots shown were cropped from full length gels for clarity. Original scans of the gels in the composite figures are shown in Supplementary Information, Figure S4. Box and whisker plots show the median, minimum and maximum values.

**Figure 2:**
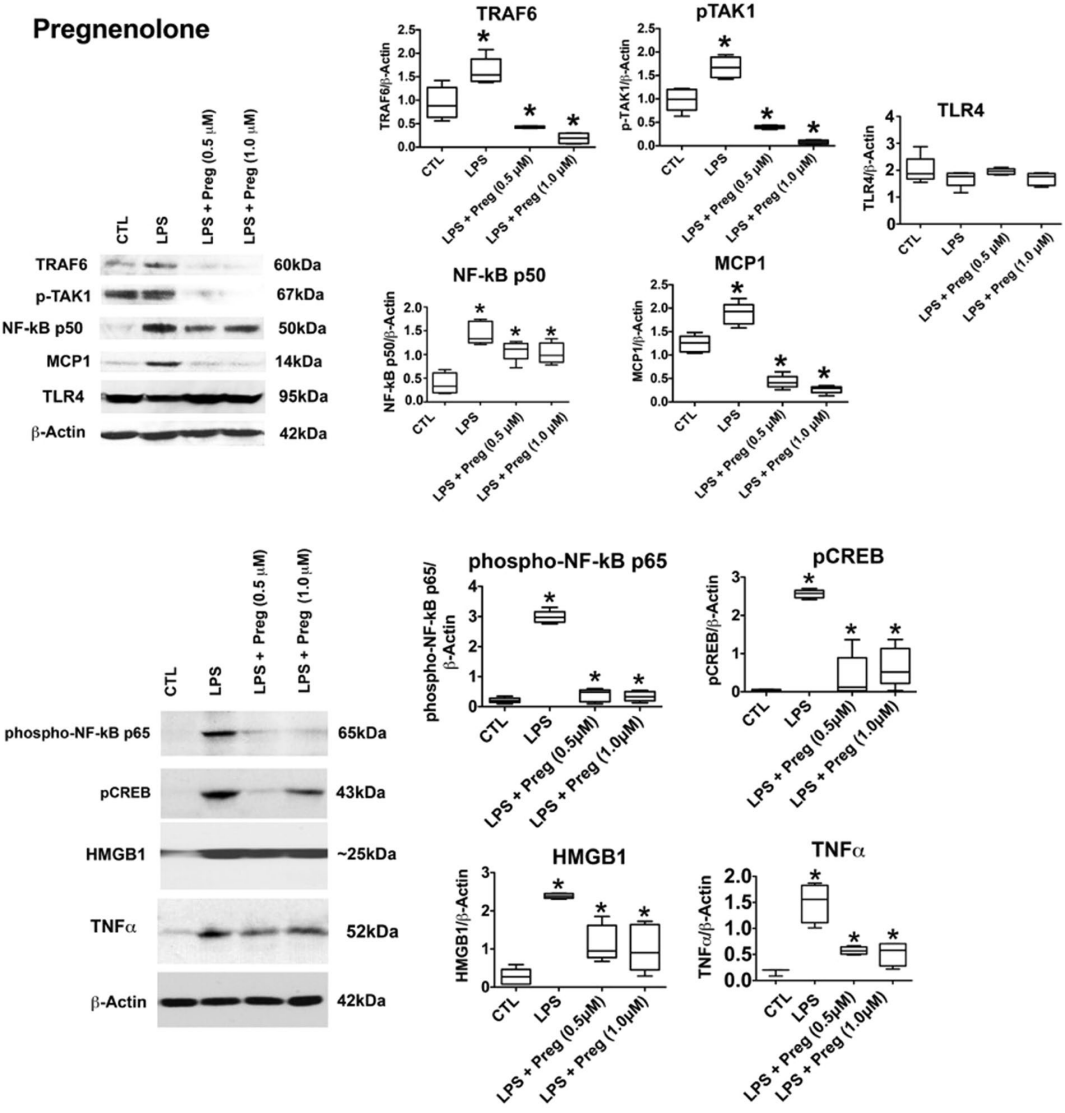
Pregnenolone inhibits LPS-activated TLR4 signaling in RAW264.7 cells. RAW264.7 cells were exposed to LPS alone (1 µg/ml) or LPS together with pregnenolone (0.5 µM or 1 µM) and harvested 24 hours later. The levels of p-TAK1 [F_19_ = 90.0, n = 5/grp], MCP-1 [F_19_ = 100.56, n = 5/grp], TRAF6 [F_19_ = 38.96, n = 5/grp], NF-κB p50 [F_19_ = 19.72, n = 5/grp], phospho-NF-κB p65 [F_19_ = 38.96, n = 5/grp], pCREB [F_19_ = 90.04, n = 5/grp], HMGB1 [F_19_ = 19.72, n = 5/grp], and TNFα [F_15_ = 25.54, n = 4/grp] were significantly increased in the LPS-treated as compared to untreated (CTL) cells but the increase was inhibited with pregnenolone (Preg) at both doses studied (*p ≤ 0.05, by One-way ANOVA; Newman-Keuls post-hoc test). Pregnenolone (0.5 µM, p = 0.1763, n = 5/grp or 1 µM, p = 0.9570, n = 5/grp) did not affect TLR4 expression. Blots shown were cropped from full length gels for clarity. Original scans of the gels in the composite figures are shown in Supplementary Information, Figure S5. Box and whisker plots show the median, minimum and maximum values.

The levels of MCP-1, p-TAK1, TRAF6, and NF-κB p50 were significantly higher in the LPS-treated than untreated cells, and these increases were blocked by 3α,5α-THP (Fig. 1) or pregnenolone (Fig. 2) at both doses. 3α,5α-THP inhibited the effect of LPS on MCP-1 by 81.5 ± 3.8% at 0.5 μM and 85.2 ± 4.5% at 1.0 μM (Fig. 1). It also inhibited the effect of LPS on p-TAK1 by 37.8 ± 7.7% at 0.5 μM and 71.7 ± 3.6% at 1.0 μM, TRAF6 by 54.5 ± 5.5% at 0.5 μM and 55.3 ± 2.6% at 1.0 μM. 3α,5α-THP did not affect TLR4 expression (Fig. 1). Pregnenolone inhibited the effect of LPS on MCP-1 by 77.3 ± 7.3% at 0.5 μM and 85.8 ± 4.4% at 1.0 μM. It also inhibited the LPS effect on p-TAK1 by 76.2 ± 2.0% at 0.5 μM and 95.2 ± 2.5% at 1.0 μM, TRAF6 by 73.7 ± 1.3% at 0.5 μM and 88.5 ± 6.8% at 1.0 μM. Pregnenolone did not affect TLR4 expression (Fig. 2) and its effects on the TLR4-activated proteins were roughly equivalent at both doses, suggesting a maximal effect was obtained at 0.5 μM (Fig. 2). 3α,5α-THP inhibited NF-κB p50 by 19.8 ± 7.9% at 0.5 μM and 38.3 ± 7.3% at 1.0 μM (Fig. 1) and pregnenolone by 25.3 ± 7.4% at 0.5 μM and 28.8 ± 6.7% at 1.0 μM (Fig. 2), potentially indicative of the contribution of other transcription factors to the neurosteroids’ effect on LPS-induced MCP-1 upregulation. LPS did not alter cell viability in the RAW264.7 cells. Cell death after mock-treatment (control) was 4.0 ± 0.88%, and cell death after LPS-treatment was 4.4 ± 0.61% (t-test, p = 0.21).

Since pregnenolone is a precursor for 3α,5α-THP, we considered the possibility that pregnenolone may have been converted in the RAW264.7 cells by analysis of 3α,5α-THP levels in the cell culture media at the time of cell harvest. 3α,5α-THP was detected at less than 0.69 ± 0.11 nmol/L, indicative of less than 0.1% conversion of 1.0 μM pregnenolone (data not shown). This result suggests that the pregnenolone effects were not due to its conversion to 3α,5α-THP.

### 3α,5α-THP and pregnenolone inhibit the LPS-induced proinflammatory response in RAW264.7 cells

Because the neurosteroids had relatively little effect on NF-κB p50, we considered the possibility that inhibition of other transcription factors and proinflammatory responses may be involved. RAW246.7 cells were treated as described above and protein extracts were immunoblotted with antibodies to phospho-NF-κB p65, pCREB, the proinflammatory cytokine tumor necrosis factor alpha (TNFα), and high mobility group box-1 (HMGB1), a highly conserved non-histone chromosomal protein, the translocation of which from the intra- to extra-cellular environment is a critical event in inflammatory responses. Indeed, HMGB1 is currently recognized as a cytokine secreted from activated macrophages and other inflammatory cells during the innate immune response and it is believed to function as a TLR4 ligand. HMGB1 binds to the LPS-activated TLR4/MD-2 complex, which initiates transduction of a signal that stimulates macrophage release of proinflammatory cytokines, including TNFα^55,56^. The data summarized in Figs 1 and 2 indicate that LPS caused a significant increase in the levels of phospho-NF-κB p65 and pCREB (p < 0.0001), but the increase was blocked by 3α,5α-THP and pregnenolone at both 0.5 μM and 1.0 μM doses. 3α,5α-THP inhibited the effect of LPS on phospho-NF-κB p65 by 90.1 ± 8.5%, p < 0.0001 at 0.5 μM and 88.9 ± 10.8%, p < 0.0001 at 1.0 μM. 3α,5α-THP inhibited the effect of LPS on pCREB by 97.2 ± 1.9%, p < 0.0001 at 0.5 μM and 94.8 ± 3.4%, p < 0.0001 at 1.0 μM. Similar to 3α,5α-THP, pregnenolone inhibited the effect of LPS on phospho-NF-κB p65 by 86.7 ± 7.3%, p < 0.0001 at 0.5 μM and 88.1 ± 5.5%, p < 0.0001 at 1.0 μM. Pregnenolone inhibited the effect of LPS on pCREB by 84.8 ± 9.9%, p < 0.01 at 0.5 μM and 83.7 ± 8.9%, p < 0.01 at 1.0 μM. Thus, both steroids were effective in inhibiting LPS activation of nuclear transcription factors that initiate the feed-forward proinflammatory signaling.

**Figure 3:**
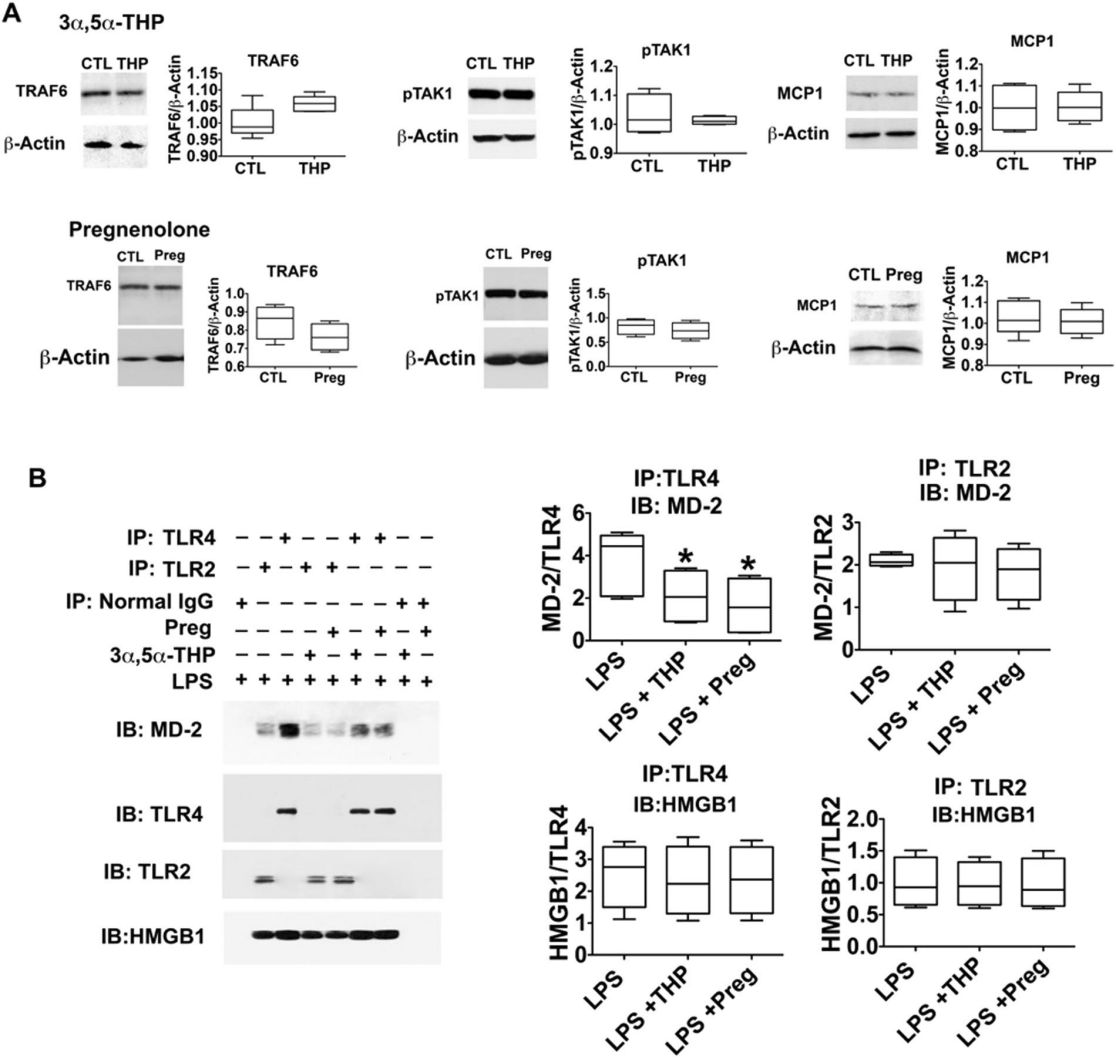
Neurosteroids target the activated TLR4 signal by inhibiting TLR4/MD-2 binding. (**A**) 3α,5α-THP and pregnenolone specifically target the activated TLR4 signal. RAW264.7 cells untreated (CTL) or treated with 3α,5α-THP (THP; 1 µM) or pregnenolone (Preg; 1 µM) were harvested after 24 hrs. The levels of p-TAK1, TRAF6, and MCP-1 were similar in the neurosteroid-treated and untreated cells, indicating that the neurosteroids specifically target only the activated TLR4 signal. **(B)** Neurosteroids inhibit TLR4 signal activation in RAW264.7 cells by blocking TLR4/MD-2 binding. RAW246.7 cells were treated with LPS (1 μg/ml) without or with 3α,5α-THP (THP; 1.0 μM) or pregnenolone (Preg; 1.0 μM) and protein extracts collected at 24 hrs post-treatment were immunoprecipitated (IP) with antibody to TLR4 or TLR2. The precipitates were immunoblotted (IB) with MD-2 antibody. Normal IgG was used as control. MD-2 co-precipitated with TLR4, but not normal IgG. The levels of MD-2 co-precipitating with TLR4 were significantly reduced by 3α,5α-THP (45.4 ± 6.9%, p < 0.05) or pregnenolone (57.2 ± 7.3%, p < 0.05), but neither 3α,5α-THP nor pregnenolone had any effect on the minimal, presumably background, TLR2/MD-2 interaction. HMGB1 co-precipitated with both TLR4 and TLR2 and its levels were not altered by the neurosteroids. Blots shown were cropped from full length gels for clarity. Original scans of the gels in the composite figures are shown in Supplementary Information Figure S6. Box and whisker plots show the median, minimum and maximum values.

The levels of HMGB1 (p < 0.0001) and TNFα (p < 0.001) were also significantly increased in the LPS-treated cells and this was inhibited by both 3α,5α-THP and pregnenolone. 3α,5α-THP inhibited the effect of LPS on HMGB1 by 88.9 ± 11.0%, p < 0.0001 at 0.5 μM and 58.6 ± 5.5%, p < 0.0001 at 1.0 μM. 3α,5α-THP inhibited the effect of LPS on TNFα by 77.8 ± 7.3%, p < 0.01 at 0.5 μM and 70.9 ± 3.5%, p < 0.01 at 1.0 μM. Similar to 3α,5α-THP, pregnenolone inhibited the effect of LPS on HMGB1 by 52.0 ± 9.8%, p < 0.01 at 0.5 μM and 57.5 ± 12.8%, p < 0.01 at 1.0 μM. Pregnenolone inhibited the effect of LPS on TNFα by 61.7 ± 3.6%, p < 0.01 at 0.5 μM and 65.1 ± 7.7%, p < 0.01 at 1.0 μM. Collectively, the data indicate that the neurosteroids have a broad range of inhibitory activity in RAW246.7 cells that is specifically centered on activated TLR4 pathways. Importantly, both 3α,5α-THP and pregnenolone (1 µM) failed to inhibit the expression of p-TAK1, TRAF6, and MCP-1 in non-activated RAW264.7 cells in the absence of LPS (Fig. 3A).

### Neurosteroids inhibit TLR4 signal activation in RAW246.7 cells by blocking TLR4/MD-2 binding

Because protein-protein interactions are known to initiate the TLR4 signaling cascade in immune cells^57–59^, we next asked whether the neurosteroids interfere with the formation of the TLR4/MD-2 complex that initiates LPS-mediated signal activation through the MyD88-dependent cascade, which includes TRAF6, p-TAK1, and the activated transcription factors leading to HMGB1, MCP-1 and TNFα^56,60^. RAW246.7 cells were treated with LPS (1 μg/ml) without or with 3α,5α-THP (1.0 μM) or pregnenolone (1.0 μM) and protein extracts were collected 24 hrs post-treatment and immunoprecipitated with antibody to TLR4. Immunoprecipitation with normal IgG and antibody to TLR2 served as controls. To measure co-precipitation, the precipitates were immunoblotted with MD-2 antibody.

In the LPS-treated cells, MD-2 co-precipitated with TLR4, but not normal IgG, indicative of TLR4/MD-2 binding (Fig. 3B). The levels of MD-2 co-precipitated with TLR4 were significantly reduced by treatment with 3α,5α-THP (45.4 ± 6.9% reduction, p < 0.05) or pregnenolone (57.2 ± 7.3% reduction, p < 0.05). In contrast, as a negative control, TLR2/MD-2 co-immunoprecipitation was not altered by either 3α,5α-THP or pregnenolone. The data indicate that both steroids selectively inhibit TLR4/MD-2 complex formation and presumably thereby, the resulting signaling pathway activation. We conclude that the inhibitory effect of the neurosteroids is specific for the TLR4/MD-2 interaction that initiates the LPS-induced HMGB1 upregulation, because immunoblotting of the precipitates with HMGB1 antibody indicated that HMGB1 co-precipitates with both TLR4 and TLR2, and these protein binding interactions are not altered by the neurosteroids (Fig. 3B).

### 3α,5α-THP inhibits TLR4 signaling and TLR4 heterodimerization in the P rat VTA

**Figure 4:**
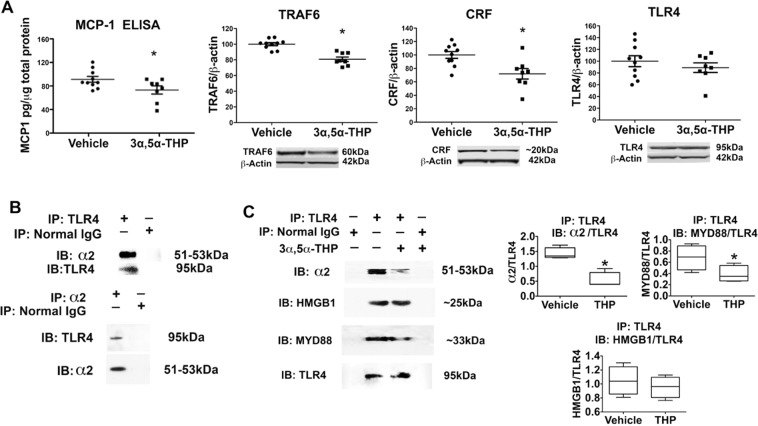
3α,5α-THP inhibits TLR4 signal innately activated in P rat VTA by blocking TLR4/α2 binding and TLR4/MyD88 binding. (**A**) 3α,5α-THP administration (15 mg/kg) significantly reduced MCP-1 (ELISA; Student’s t(16) = 2.19), TRAF6 (Student’s t(16) = 5.74), and CRF (Student’s t(16) = 3.112) levels compared to vehicle controls, with no effect on TLR4 protein expression. *p < 0.05 compared to control. **(B)** TLR4 binds α2 in the P rat VTA. Protein extracts from P rat VTA were immunoprecipitated (IP) with the TLR4 or α2 antibodies or normal IgG (control) and the precipitates were reciprocally immunoblotted (IB) with α2 or TLR4 antibodies. Both α2 and TLR4 were seen in the anti-α2 and anti-TLR4 (but not normal IgG) precipitates from P rat VTA, indicative of protein-protein interaction. **(C)** 3α,5α-THP inhibits TLR4/α2 and the downstream TLR4/MyD88 binding in the P rat VTA. Protein extracts obtained from P rat VTA after 3α,5α-THP (15 mg/kg) or vehicle control administration were immunoprecipitated (IP) with antibody to TLR4. The precipitates were immunoblotted (IB) with α2 antibody. Normal IgG was used as control. α2 co-precipitated with TLR4, but not normal IgG. The levels of α2 co-precipitating with TLR4 were significantly reduced by 3α,5α-THP (62.7 ± 9.2% reduction, p < 0.001). 3α,5α-THP also inhibited the binding of TLR4 to MyD88 (43.5 ± 5.4% inhibition, p < 0.05). HMGB1 bound TLR4, but binding was not altered by 3α,5α-THP. Blots shown were cropped from full length gels for clarity. Original scans of the gels in the composite figures are shown in Supplementary Information Figure S7. Scatter plots show the mean and S.E.M. of values. Box and whisker plots show the median, minimum and maximum values.

To examine whether the neurosteroids also inhibit TLR4 signal activation in the brain, selectively bred P rats that have an innately activated TLR4 signal in the VTA^45^, were administered 3α,5α-THP (15 mg/kg, IP) or pregnenolone (75 mg/kg, IP), sacrificed after 45 minutes and examined for TLR4 signaling using parallel measures. Since the CRF/CRFR1 system is also associated with alcohol drinking^49,50,52,61^, and CRF was shown to sustain the activated TLR4 signal, also in the P rat VTA^41^, the effects of the neurosteroids on CRF were studied in parallel. 3α,5α-THP administration reduced the levels of MCP-1 by 20 ± 9% (p < 0.05), TRAF6 by 19 ± 3% (p < 0.0001), and CRF by 28 ± 9% (p < 0.01), with no effect on TLR4 protein expression (Fig. 4A). Pregnenolone administration had no effect on TRAF6, CRF, or TLR4 (data not shown).

Since previous studies had shown that the activated TLR4 signal is downstream of the GABA_A_R α2 subunit in P rat brain (Liu *et al*.^45^) and GABA_A_R α2 binds TLR4 in Neuro2A cells (Balan *et al*., 2018), we first investigated if TLR4 co-precipitates with the GABA_A_R α2 subunit in P rat VTA. Protein extracts were immunoprecipitated with antibody to TLR4, followed by immunoblotting with α2 antibody. Immunoprecipitation with normal IgG served as control. As shown in Fig. 4A, α2 co-precipitated with TLR4, but not normal IgG, and binding was confirmed by precipitation with GABA_A_R α2 antibody and immunoblotting with TLR4 antibody. Next, co-immunoprecipitation studies were conducted in the VTA from the P rats treated with vehicle (45% w/v 2-hydroxypropyl-β-cyclodextrin) or 3α,5α-THP (15 mg/kg, IP), to determine if 3α,5α-THP alters TLR4 interaction with GABA_A_R α2, MyD88 or HMGB1. Figure 4C shows that TLR4/GABA_A_R α2 co-immunoprecipitation is inhibited by 3α,5α-THP (62.7 ± 9.2%, p < 0.001). Full-length gels are shown in SI, Figure S3. Interestingly, TLR4/MyD88 interaction was also inhibited by 3α,5α-THP (43.5 ± 5.4%, p < 0.05) (Fig. 4C), suggesting that 3α,5α-THP may bind TLR4 in a manner that affects its interaction with both GABA_A_R α2 and MyD88. HMGB1 also bound TLR4, but binding was not altered by 3α,5α-THP (Fig. 4B). To our knowledge, this is the first report that TLR4 binds the GABA_A_R α2 subunit in the brain resulting in TLR4 signal activation, and neurosteroids inhibit this innate activation mechanism. However, the precise site of neurosteroid interactions with the proteins, and the full spectrum of protein-protein interactions remain unknown.

## Discussion

**Figure 5:**
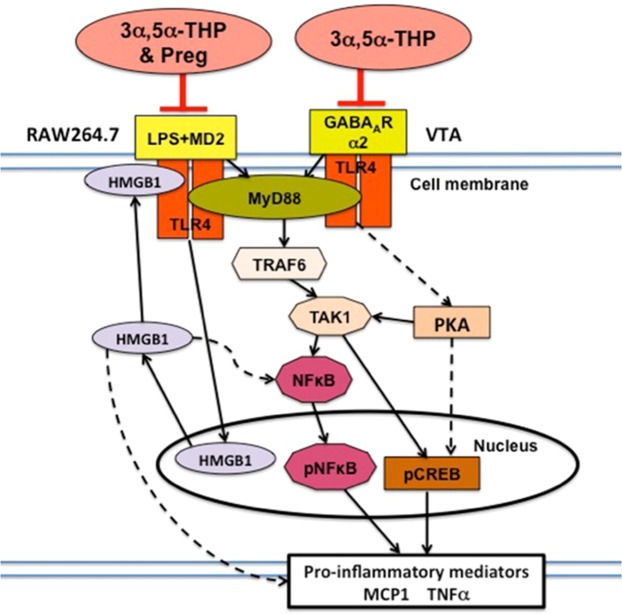
Schematic of activated TLR4 signal inhibited by neurosteroids. LPS and GABA_A_R α2, respectively activate the TLR4 signal in RAW246.7 cells and P rat VTA. Signal activation initiates with LPS-induced TLR4/MD-2 complex formation at the cell surface in RAW246.7 cells and TLR4/GABA_A_R α2 or TLR4/MyD88 complex formation in the P rat VTA. Complex formation is followed by the intracellular signal, one direction of which is the (MyD88)-dependent pathway that activates TRAF6 and TAK1 and results in the activation (phosphorylation) of the transcription factors NF-κB and CREB. An alternate pathway activates PKA/CREB^99^. Activated (phosphorylated) transcription factors translocate to the nucleus and initiate the production of various proinflammatory mediators, including TNFα. 3α,5α-THP inhibits both the LPS/TLR4/MD-2 and α2/TLR4 complex formation and pregnenolone (Preg) inhibits the LPS/TLR4/MD-2 complex formation and thereby, both inhibit resulting intracellular signaling. The LPS-stimulated TLR4/MD-2 interaction also initiates the ability of LPS to increase HMGB1 expression, and this is also inhibited by 3α,5α-THP and pregnenolone in RAW246.7 cells, apparently through inhibition of the TLR4/MD-2 complex formation. Released HMGB1 can bind TLR4 or/and modulate the production of proinflammatory mediators through NF-κB-dependent or NF-κB-independent signaling pathways (dashed lines)^56,60,62,100^.

These studies provide direct evidence for 3α,5α-THP and pregnenolone-mediated inhibition of TLR4 signal activation in monocyte/macrophage (RAW246.7) cell cultures and 3α,5α-THP inhibition in the VTA of alcohol-preferring P rats. We further document their action at the initiating protein-protein interaction event, as schematically represented in Fig. 5.

In RAW264.7 cells, the TLR4 agonist LPS increased the levels of p-TAK1; TRAF6; transcription factors NF-κB p50, phospho-NF-κB p65, and pCREB; and the proinflammatory mediators, HMGB1, MCP-1, and TNFα. All of these effects were inhibited by both neurosteroids at 0.5 and 1.0 μM doses. Neurosteroid-mediated inhibition was specific for the activated pathways and was not seen in the non-LPS treated cells. Inhibition appeared to involve the ability of 3α,5α-THP and pregnenolone to block the binding of TLR4 to MD-2, suggesting that both steroids interfere with the initiating step of the LPS-mediated TLR4 signal activation. However, we cannot rule out the possibility that the neurosteroids’ function at the TLR4 signal activation step involves interaction with additional members that contribute to TLR4 oligomerization, including LPS itself, LPS binding protein (LBP) or CD-14. Further studies are needed to address this question.

Pregnenolone is a precursor of 3α,5α-THP in steroidogenic cells, but we found no evidence of the conversion of pregnenolone to 3α,5α-THP in the media of RAW264.7 cells. Thus, it is likely that pregnenolone inhibition of TLR4 signaling in RAW264.7 cells is due to an intrinsic property of the steroid. Further, pregnenolone produced maximal effects at lower doses than 3α,5α-THP in the RAW264.7 cells, suggesting that it may have greater inhibitory efficacy in the TLR4 signaling pathway. The ability of both 3α,5α-THP and pregnenolone to block the binding of TLR4 to MD-2 may be related to their identical structures in the steroid D ring. However, further studies will be needed to assess the structural requirements for steroid inhibition of TLR4 signaling in macrophages.

The ability of the neurosteroids to inhibit the LPS-induced upregulation of HMGB1, apparently through inhibition of the TLR4/MD-2 complex formation is particularly interesting, as it provides novel information on the neurosteroid activity as well as the role of TLR4 in the regulation of HMGB1 expression. HMGB1 is a DNA-binding intranuclear protein, but recent studies have shown that it is an actively secreted cytokine produced by inflammatory cells during innate immune responses, placing HMGB1 at the intersection between the inflammatory responses of activated and non-activated inflammatory signals. In this context, LPS, the canonical TLR4 ligand, is recognized as an established HMGB1 inducer. However, the exact signaling pathway responsible for the LPS effect on HMGB1 and its contribution to the inflammatory response are still poorly understood. This appears to involve HMGB1 binding to TLR4/MD-2 and results in the transduction of a signal that stimulates macrophage release of TNFα. Both binding and signaling require the redox-sensitive cysteine in position 106^60^ and the signaling activates the nuclear translocation of activated NF-κB^62^. However, LPS and HMGB1 signaling differ. HMGB1 binds to TLR4 with much less affinity than LPS, and it activates gene expression patterns that are distinct from the LPS-mediated expression pattern^60,62,63^. Our data are consistent with these results in that the neurosteroids inhibit the LPS-induced TLR4/MD-2 interaction and HMGB1 upregulation. However, we find that they do not interfere with the ability of HMGB1 to bind both TLR4 and TLR2, suggesting that they regulate HMGB1 production, but not its function through TLR4 receptor binding.

In the VTA of alcohol-preferring P rats, 3α,5α-THP inhibited several components of the TLR4 signaling pathway including TRAF6 and MCP-1, as well as CRF, consistent with the data from the cultured macrophage cells. However, unlike the macrophage findings in which 3α,5α-THP inhibited the canonical initiating TLR4/MD2 oligomerization step, in the VTA 3α,5α-THP inhibited TLR4 oligomerization with both the GABA_A_R α2 subunit and MyD88, suggesting that it also blocks the distinct steps that initiate TLR4 signal activation in neurons. Interestingly, pregnenolone did not inhibit TRAF6 or CRF, suggesting that structural requirements for inhibition of TLR signaling are cell type specific, and likely related to the requirements of the binding partners – both TLR4 and GABA_A_R α2 subunits. Inhibition of TLR4-GABA_A_R α2 binding may require both the structure of the steroid D ring common to 3α,5α-THP and pregnenolone for TLR4 binding, as well as the A ring structure of the GABAergic neuroactive steroids for GABA α2 subunit binding^6,53^. TLR4-GABA_A_R α2 subunit co-immunoprecipitation has previously been demonstrated in Neuro 2A cells^64^, where other GABA_A_ subunits and GABA are not present, documenting the specificity of this interaction. Inhibition of TLR4-GABA_A_ α2 subunit binding may explain the inhibitory activity of 3α,5α-THP in P rat VTA, and the lack of effect of pregnenolone. While pregnenolone lacks GABAergic activity, and failed to block TRAF6 or CRF, it is possible that it may interfere with TLR4/MyD88 binding and/or the PKA - pCREB pathway in the VTA. These possibilities are presently under investigation.

3α,5α-THP has potent actions at synaptic and extrasynaptic GABA_A_ receptors^5,6^ and inhibits stress-induced hypothalamic CRF^8,9,65^. It is apparent that GABAergic inhibition is not required for the neurosteroid effects on MyD88-dependent TLR4 signaling in RAW264.7 cells, as pregnenolone mimicked the effects of 3α,5α-THP and 3α,5α-THDOC failed to inhibit TRAF6 in both macrophages and VTA (unpublished data). Moreover, 3α,5α-THP reduced CRF in the VTA, and CRF has been shown to induce TLR4 in the VTA^41^ and in macrophages^46^. This is the first demonstration that 3α,5α-THP inhibits extra-hypothalamic CRF expression and this pathway may contribute to its ability to reduce TLR4 signaling. Further studies are needed to explore this possibility.

3α,5α-THP and pregnenolone inhibition of TLR4 signaling in the periphery and 3α,5α-THP inhibition of TLR4 signaling the brain, likely contribute to the therapeutic actions of these compounds. It is well established that immune signaling via macrophages in the periphery affects brain function and may participate in the feed-forward activation of neuroimmune signaling in the brain^36,37,39^. Thus, in order to establish that TLR4 signaling is actually inhibited in the brain itself, it is critical to confirm that inhibition occurs in the absence of peripheral immune activation by LPS, stress or alcohol exposure. To this end, we studied the male P rat animal model that exhibits innate TLR4 activation in brain^41,42^ and our data indicate that 3α,5α-THP does in fact inhibit this TLR4 brain signal. However, this approach also has limitations, including the fact that innate TLR4 activation in P rats was found in neurons, so the possible inhibition of glial activation was not addressed in these studies. Furthermore, innate TLR4 activation may involve select components of proinflammatory signaling that are unique to this animal model and its function in female animals is still unknown. Further studies are needed to better understand 3α,5α-THP inhibition of TLR4 activation in brain induced by stress, alcohol and LPS in both male and female animals.

Pregnenolone and 3α,5α-THP are synthesized in the adrenals, gonads, and neurons, including brain synthesis independent of peripheral precursors^66^. Neurosteroids, like immune factors, circulate in the bloodstream, cross the blood brain barrier and diffuse between different cell types due to their lipophilic characteristics, exhibiting paracrine effects in many cells, and may affect neuroimmune signaling at the level of macrophages, neurons, or glial cells. However, neuroimmune signaling differs in these cell types^67^, consistent with the differential effects of neurosteroids in macrophages and brain. The ability of the neurosteroids to also inhibit innately activated signaling by other TLRs in native immune, neuronal, glial and endothelial cells, if present, is still unknown.

Neuroimmune signaling through TLR receptors is activated in alcohol use disorders^25,26,39^, other addictions^27^, depression^28,29^, epilepsy^30^, trauma of stroke^68^, traumatic brain injury^23,24^, Alzheimer’s Disease^69^, and multiple sclerosis^70^. Further, 3α,5α-THP has shown efficacy against seizures^71,72^, alcohol reinforcement and consumption^10,11,73,74^, cocaine craving and stress-induced craving^18,19^, schizophrenia^75^, depression^20^, traumatic brain injury^12,16^, multiple sclerosis^13^, and Alzheimer’s disease^76^. Our findings suggest that inhibition of TLR signaling may contribute to the therapeutic actions of neurosteroids in these conditions, all of which exhibit TLR4 activation and inflammation in the brain. Furthermore, this work may inform the development of novel neuroactive steroids under development for treatment of various neurological and psychiatric disorders to ensure efficacy comparable to or better than the endogenous steroids.

Despite current disagreement about which TLRs are most important in various species^77^, TLRs, particularly TLR4, are activated with binge alcohol consumption and addiction and male vs. female differences were reported^41,78–80^. Systemic injection of the TLR4-specific ligand LPS increases voluntary alcohol consumption in mice, and human alcoholics have elevated levels of plasma LPS and TLR4 activation markers^81–86^. In this context, it is also important to point out that pharmacologic and genetic studies have shown that alcohol induces CRF signaling and CRF plays a significant role in alcohol addiction^48–52,61^. CRF is known to activate or enhance TLR4 signaling and it sustains the innately activated TLR4 signal in P rats^41,46,47^. Thus, the data presented here may be particularly relevant for neurosteroid actions in the context of TLR activation by stress and/or alcohol addiction, conditions that are often co-morbid with depression, post-traumatic stress, and seizures.

In conclusion, inhibition of proinflammatory neuroimmune signaling has great promise in the treatment of several chronic neuropsychiatric diseases. Nonetheless, neuroimmune signaling has important protective as well as deleterious effects under various conditions and the appropriate balance is needed for optimal brain and immune function^87–90^. The present data suggest a novel mechanistic role for 3α,5α-THP in these processes, representing a new mechanism of action of this steroid. Thus, combined with potent activity on GABA_A_ receptors and the inhibition of CRF signaling, 3α,5α-THP inhibition of TLR4 activation in the periphery and brain may provide a novel strategy to address inflammatory disease and its effects on the brain.

## Materials and Methods

### Cells and reagents

Mouse monocyte macrophage cells (RAW264.7) that innately express TLR4 were obtained from American Type Culture Collection (Manassas, VA, USA). The cells were grown in Dulbecco’s modified Eagle’s medium (DMEM) (Gibco; Gaithersburg, MD, USA) supplemented with 10% fetal bovine serum (FBS, Gemini, West Sacramento, CA, USA), 1% penicillin/streptomycin 100× (Gibco) at 37 °C in a 5% CO_2_ humidified atmosphere and refreshed with media lacking serum 16 hrs prior to experimentation. The TLR4-specific ligand LPS (1 μg/ml) alone (Sigma-Aldrich, St. Louis, MO, USA; Cat. # L3024) or LPS (1 μg/ml) together with 3α,5α-THP (0.5 μM, 1.0 μM) or pregnenolone (0.5 μM, 1.0 μM) or 3α,5α-THP/pregnenolone alone were added to the cultures 24 hrs before cell collection. This time point was chosen for the maximal LPS effect on TLR4 signal activation in prior studies^91,92^, a finding confirmed by us (data not shown).

### Antibodies

The following antibodies were commercially obtained and used as previously described^43^. Rabbit anti-TRAF6 (AB_793346), mouse anti-NF-κB p50 (AB_628015), mouse anti-TNFα (AB_630341), mouse anti-TLR2 (AB_628364), and mouse anti-TLR4 (AB_10611320) were from Santa Cruz Biotechnology (Santa Cruz, CA, USA). Rabbit phospho-TAK1 (Ser412) (p-TAK1) (AB_2140096), mouse phospho-NF-κB p65 (Ser536) (AB_331281), rabbit phospho-CREB (Ser133) (AB_2561044) were from Cell Signaling Technology (Danvers, MA, USA). Mouse anti-CCL2 (MCP-1) (AB_2538512), and rabbit anti-MD-2 (AB_11155832) were from Thermo Fisher Scientific (Waltham, MA, USA). Rabbit-derived GABA_A_ α2 antibody (amino acids 322–357) was provided by W. Sieghart (Center for Brain Research, Medical University of Vienna; Vienna; Austria; AB_2532077). Mouse anti-beta-Actin (β-Actin) (AB_2687938), and rabbit anti-HMGB1 (AB_2232989) were from Proteintech Group (Rosemont, IL, USA), rabbit anti-MyD88 (AB_2722690) from NeoScientific (Woburn, MA, USA), and rabbit anti-CRF (AB_2314240) from Peninsula Labs (San Carlos, CA, USA). Horseradish peroxidase-labeled secondary antibodies were anti-rabbit IgG (AB_2099233) and anti-mouse IgG (AB_330924) from Cell Signaling Technology.

### Immunoblotting

The assay used for RAW264.7 cell lysates and co-immunoprecipitation was as previously described^41,42,45^. Total protein was determined by the bicinchoninic acid assay (BCA, Thermo Fisher Scientific, Waltham, MA, USA, Cat.# 23228 and Cat.# 1859078). The proteins (100 μg/lane) were separated by SDS–polyacrylamide gel electrophoresis, transferred to polyvinylidene fluoride membranes (PVDF, Bio-Rad, Cat.# 162–0177), blocked with 5% Blotting-Grade Blocker (Bio-Rad, Cat. # 1706404) or 5% BSA (for phosphorylated primary antibodies) and exposed to primary antibody overnight (4 °C), followed by horseradish peroxidase-labeled secondary antibodies. Immunoreactive bands were visualized with the Plus-ECL kit reagents (Perkin Elmer, Waltham, MA, USA, Cat.# NEL105001EA) followed by exposure to high-performance chemiluminescence film (Hyperfilm ECL; Amersham). Quantitation was by densitometric scanning with a Bio-Rad GS-700 imaging densitometer. Blots were stripped and re-probed with different primary antibodies 3–5 times. Each densitometric measurement was divided by the corresponding β-Actin densitometric measurement and the results [n = 5/group] are expressed as the mean β-Actin-adjusted densitometric units ± SEM.

Immunoblotting for whole VTA lysates was done as previously described^93^. Briefly, VTA micropunches (1 mm thick) were lysed with CelLytic MT (Sigma-Aldrich) and protease and phosphatase inhibitor cocktail. Total protein was determined by the BCA assay. The proteins (10 μg/lane) were resolved by NuPAGE™ 4–12% Bis-Tris Midi Protein Gel (Thermo Fisher, Waltham, MA) electrophoresis and transferred using the iBlot 2 Dry Blotting System (Thermo Fisher, Waltham, MA). Blots were exposed to antibodies and normalization was with β-actin. Detection was with enhanced chemilumnesence (GE Healthcare, Amersham, UK). Membranes were exposed to film under non-saturating conditions. Densitometric analysis was conducted using NIH Image 1.57.

Antibody validation was previously described^43^ and specific protein detection using full-length gels is shown for RAW246.7 cells [untreated (CTL), LPS-treated (LPS), and treated with LPS together with 3α,5α-THP] and for the VTA, in Supplementary Information (SI), Figures S1–2.

### Co-Immunoprecipitation Assay

RAW264.7 cells [treated with LPS alone (1 μg/ml) or together with 3α,5α-THP (1 μM) or pregnenolone (1 μM) for 24 hrs] were exposed to chemical protein crosslinking with membrane-permeable DSP (1 mM,Thermo Fisher Scientific, Cat. # PG82081) for 20 min on ice as previously described^94^. The crosslinker was quenched in 1 M Tris buffer (pH 7.5), proteins were centrifuged at 21,000 × g for 15 min and extracted with Pierce IP Lysis Buffer (Thermo Fisher Scientific, Cat. # 87787) supplemented with protease and phosphatase inhibitor cocktails (Sigma). VTA micropunch proteins were extracted with CelLytic MT (Sigma Aldrich, St. Louis, MO, USA, Cat. # C3228) supplemented with protease and phosphatase inhibitor cocktails (Sigma) and co-immunoprecipitation was conducted as previously described^43,95^. Proteins were resolved by SDS-polyacrylamide gel electrophoresis, transferred to PVDF membranes and immunoblotted with MD-2, HMGB1, MYD88, GABA_A_R-α2, TLR2, or TLR4 antibodies. The full-length gels showing specific TLR4/MD-2 protein interaction in RAW246.7 cells and TLR4/α2 and TLR4/MyD88 interaction the VTA are shown in SI, Figure S3.

### 3α,5α-THP Radioimmunoassay (RIA)

3α,5α-THP concentrations in the RAW264.7 cell media were measured by radioimmunoassay as described elsewhere^96^, modified for use with cell media^10^. Briefly, 3α,5α-THP was extracted from cell media three times with 3 ml of ethyl acetate and spiked with 1000 counts per minute of [^3^H]3α,5α-THP for recovery. The extracts were purified using solid phase silica columns (Burdick and Jackson, Muskegon, MI) and used for the assay (run in duplicate) and for recovery measurement. Steroid levels in the samples were extrapolated from a concurrently run standard curve and corrected for their respective extraction efficiencies. The 3α,5α-THP antibody (1:500) was provided by the late Dr. Robert Purdy at Scripps Research Institute. Antibody specificity was previously verified and no significant cross reactivity with pregnenolone, progesterone, pregnanolone or 3α,5α-THDOC was found. The validity of the assay has been verified by gas chromatography mass spectrometry determinations^97^. 3α,5α-THP values are expressed as ng/ml of cell media.

### Cell Viability

To determine if LPS exposure for 24 hrs altered cell viability, an aliquot of the cell suspension was incubated with an equal volume of 0.4% trypan blue and percentage of dead cells (identified by blue staining) was calculated relative to the total cell numbers in four independent fields using a hemacytometer.

### Animals

Selectively bred, but alcohol naïve Alcohol-preferring (P) rats (male, 3–4 months old; 250–550 g; n = 7–9/group) were obtained from the Alcohol Research Center, Indiana University School of Medicine. Male P rats were examined due to innate activation of neuronal TLR4 signaling in brain^41,42^ that allowed us to examine inhibition of TLR signaling without activation of the peripheral immune response. Animals were double housed in Plexiglas cages containing corn cob bedding and food and water was available *ad libitum*. The colony room was maintained on a normal 12 hr light-dark cycle (light onset at 0700 hr). Procedures followed National Institutes of Health Guidelines under UNC Institutional Animal Care and Use Committee approved protocols at University of North Carolina School of Medicine. Rats were habituated to handling for 7 days prior to administration of 3α,5α-THP (15 mg/kg, IP), pregnenolone (75 mg/kg, IP), 3α,5α-THDOC (15 mg/kg, IP), or vehicle (45% w/v 2-hydroxypropyl-β-cyclodextrin) and returned to their home cage. Rats were sacrificed after 45 minutes and the brain was removed and frozen at −80 °C until VTA micropunches were collected from 1 mm cryostat brain sections. This time point was selected because 3α,5α-THP is rapidly metabolized *in vivo*^53^, but has behavioral and pharmacological activity at this time point^98^.

### ELISA

VTA micropunches were lysed with CelLyte MT and the extracts were assayed for protein content by the BCA procedure (Pierce) and for MCP-1 using the rat MCP-1 ELISA kit (Raybiotech, Cat. #ERC-MCP-1-CL; Norcross, GA, USA) as per manufacturer’s instructions.

### Statistics

Measures in the RAW264.7 cells were analyzed using a one-way analysis of variance (ANOVA) followed by the multiple comparison Student-Newman-Keuls test, with p < 0.05 considered statistically significant, n = 5–8/group. In the VTA micropunches, values were analyzed by Student’s t-test for comparison of 2 groups, with n = 8/group. Analyses were performed using Graphpad Prism 5.0. Statistical details are given in the Figure Legends.

## Supplementary information

Supplementary Info

## Data Availability

All data are available upon reasonable request.
