# Supplementary material for: Endogenous Neurosteroid (3α,5α)3-Hydroxypregnan-20-one Inhibits Toll-like-4 Receptor Activation and Pro-inflammatory Signaling in Macrophages and Brain
Source: Sci Rep. 2019 Feb 4;9:1220. doi: 10.1038/s41598-018-37409-6 (PMC6362084; doi:10.1038/s41598-018-37409-6)
Supplement: Supplementary file 1 — Supplementary Info [file 41598_2018_37409_MOESM1_ESM.pdf]

SUPPORTING INFORMATION (SI)

Endogenous Neurosteroid (3α,5α)3-Hydroxypregnan-20-one Inhibits Toll-like-4 Receptor Activation and Pro-inflammatory Signaling in Macrophages and Brain

Irina Balan[1], Matthew C. Beattie[2], Todd K. O'Buckley[2], Laure Aurelian[1,3] and A. Leslie Morrow[2,3]

Running Title (50 letters): SI: Neurosteroid Inhibition of Neuroimmune Signaling

Affiliations:

1: Department of Pharmacology, University of Maryland School of Medicine, Baltimore, Maryland
2: Department of Psychiatry and Pharmacology, Bowles Center for Alcohol Studies, University of North Carolina at Chapel Hill, Chapel Hill, North Carolina
3: Contributed Equally

<u>Antibody validation and confirmation of protein identity in RAW246.7 cells.</u> The FDA definition of validation is “the process of demonstrating, through the use of specific laboratory investigation, that the performance characteristics of an analytical method are suitable for its intended analytical use” (www.fda.gov/downloads/Drugs/GuidanceComplianceRegulatoryInformation/Guidances/UCM070107.pdf).
For antibodies, validation requires demonstration that they are specific, selective, and reproducible. Immunoblotting is the widely used method to determine antibody specificity and confirmation of protein identity. It involves detection of a single band at the known molecular weight for the target. The key to proving specificity of the detected proteins by validated antibody is the visualization of full-length SDS gels and the use of appropriate controls. Increased protein expression by cell treatment with specific ligands provides positive control while reduced expression (signal >25% weaker) is a negative control.

The current studies are designed to examine inhibition of the TLR4 signal. The TLR4 antibody pivotal to these efforts was previously validated[1-4]. The antibody (Cat. #sc-293072) was obtained from Santa Cruz Biotechnology (Santa Cruz, CA, USA). It is a monoclonal antibody, gG_1 (kappa light chain) raised against amino acids 198-395 of mouse TLR4. It is documented to recognize the mouse, rat, human and canine protein by immunoprecipitation, immunoblotting, immunohistochemistry and immunofluorescence and has a total of 45 citations of its specific use (https://www.scbt.com/scbt/product/tlr4-antibody-25?requestFrom=search). The control antibody is to β-actin (Proteintech, Rosemont, IL, USA, Cat. # 66009-1-Ig) and immunoblotting is done as described in the Material and Methods (M&M) section of the manuscript. Here, we show that the TLR4-specific activating ligand LPS, increases the expression of the signal members TRAF6, pTAK1, NF-kB p50, MCP-1, pNF-kBp65, pCREB, HMGB1, and TNF-α, but not TLR4, in RAW246.7 cells and this is inhibited by 3α,5α-THP. The full-length gels, which encompass molecular weights 180-17kDa, document specificity (Fig. S1). Quantitation is shown for these proteins in the Results section of the manuscript, Figs. 1 and 2.

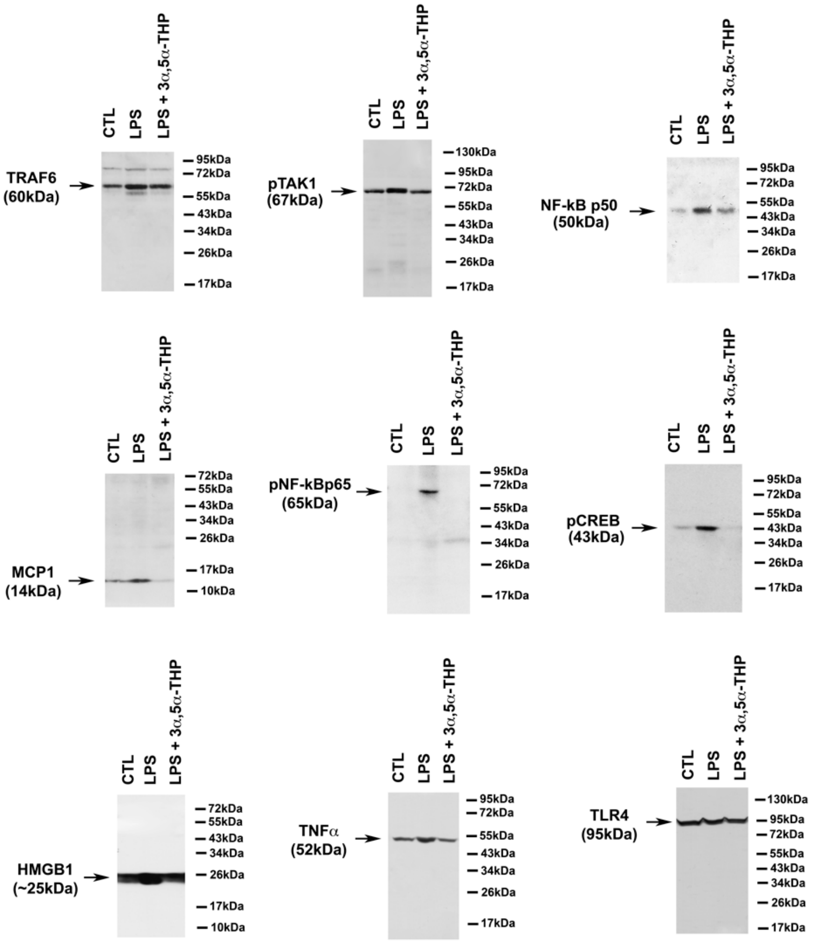

Fig. S1. Neurosteroids inhibit LPS-induced TLR4 activation. RAW246.7 cells were untreated (control, CTL) or treated with LPS alone (1 μg/ml) or LPS together with 3α,5α-THP (0.5 μM) and protein extracts collected at 24 hrs post treatment were separated by SDS–polyacrylamide gel electrophoresis and immunoblotted with antibodies to TRAF6, pTAK1, NF-κB p50, MCP1, phospho-NF-kB p65 (pNF-kB p65), pCREB, HMGB1, TNFα, or TLR4 as described in the manuscript. The full length gels confirm the specificity of the protein detection and indicate that the levels of TRAF6, pTAK1, NF-κB p50, MCP1, pNF-kB p65, pCREB, HMGB1 and TNFα, (but not TLR4) were significantly increased in LPS treated vs. untreated (CTL) cells, and inhibited by 3α,5α-THP.

Confirmation of protein identity in P rat ventral tegmental area (VTA) micropunches: Effects of systemic administration of 3α,5α-THP (15 mg/kg, IP) to P rats. To determine the effects of 3α,5α-THP (15 mg/kg, IP) on innate activation of the TLR4 signal, VTA micropunches were collected from rats 45 minutes following 3α,5α-THP or vehicle administration. Here we show the effects on TRAF6, CRF, and TLR4 protein expression, with β-actin expression included as a control. Figure S2 shows representative full length gels that encompass molecular weights 198-14kDa for each protein to document specificity of the representative blots shown in the manuscript figures.

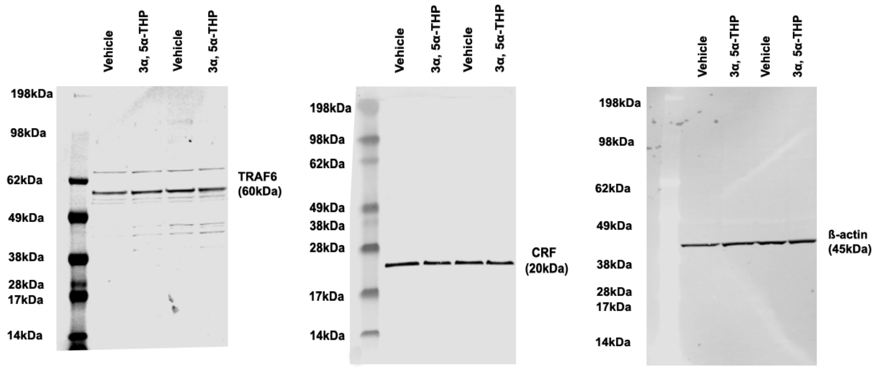

Fig. S2. Neurosteroids inhibit LPS-induced TLR4 activation. **(A)** 3α,5α-THP administration (15mg/kg, IP) significantly reduced TRAF6 (Student's t(16)=5.74), and CRF (Student's t(16)=3.112) levels compared to vehicle controls. All signals were normalized to β-actin and compared to control values for quantitation as described in the Methods section of the manuscript.

Confirmation of protein identity and protein-protein interactions. Here we provide further evidence that the TLR4 protein is expressed in RAW246.7 cells where it specifically interacts with the MD-2 protein (Fig. S3A,B), and in the VTA where it specifically interacts with the $GABA_AR$ α2 and MyD88 proteins (Fig. S3C-E). This is shown in the full-length gels of immunoprecipitation/immunoblotting (IP/IB) assays and the absence of non-specific bands.

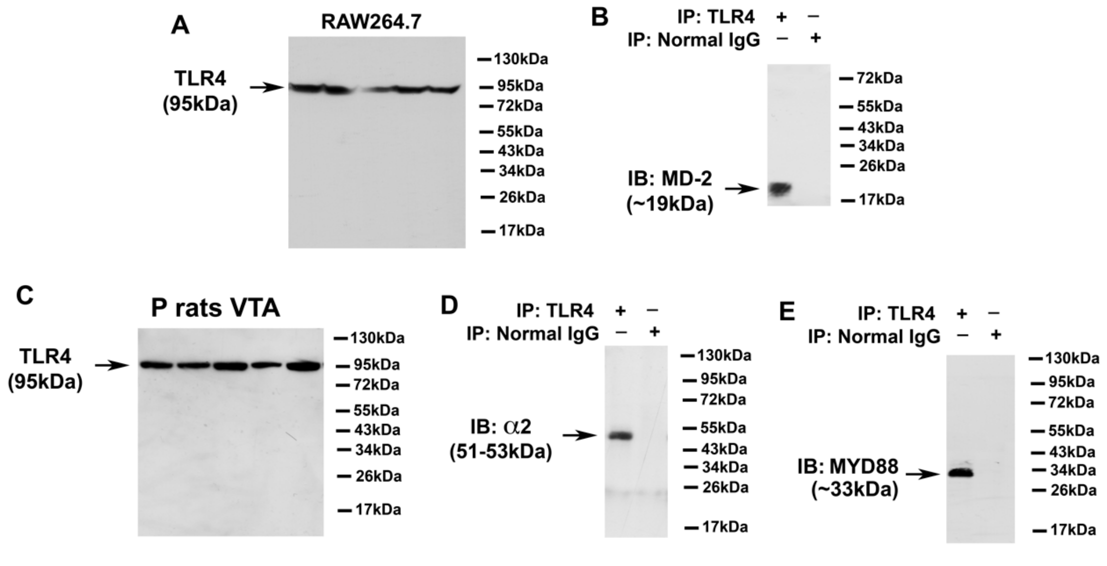

Fig. S3. TLR4 protein interactions in RAW246.7 cells and the VTA. (A) Protein extracts from RAW264.7 cells treated with LPS (1μg/ml) for 24 h (n=5) were immunoprecipitated (IP) and immunoblotted (IB) with TLR4 antibody. **(B)** Protein extracts from RAW264.7 cells treated with LPS (1μg/ml) for 24 h were immunoprecipitated (IP) with TLR4 antibody or normal IgG (control) and immunoblotted (IB) with MD-2 antibody. MD-2 was seen in the anti-TLR4 (but not normal IgG) precipitates, indicative of protein-protein interaction. **(C)** Protein extracts from P rats VTA (n=5/grp) were immunoprecipitated (IP) and immunoblotted (IB) with TLR4 antibody. **(D)** Protein extracts from P rats VTA were immunoprecipitated (IP) with TLR4 antibody or normal IgG (control) and immunoblotted (IB) with $GABA_AR$ α2 antibody. $GABA_AR$ α2 was seen in the anti-TLR4 (but not normal IgG) precipitates, indicative of protein-protein interaction. **(E)** Protein extracts from P rats VTA were immunoprecipitated (IP) with TLR4 antibody or normal IgG (control) and immunoblotted (IB) with MyD88 antibody. MyD88 was seen in the anti-TLR4 (but not normal IgG) precipitates, indicative of protein-protein interaction. The full-length gels document specific detection.

Unprocessed Original Scans of the Blots Used in All Composite Figures

Manuscript Figure 1:

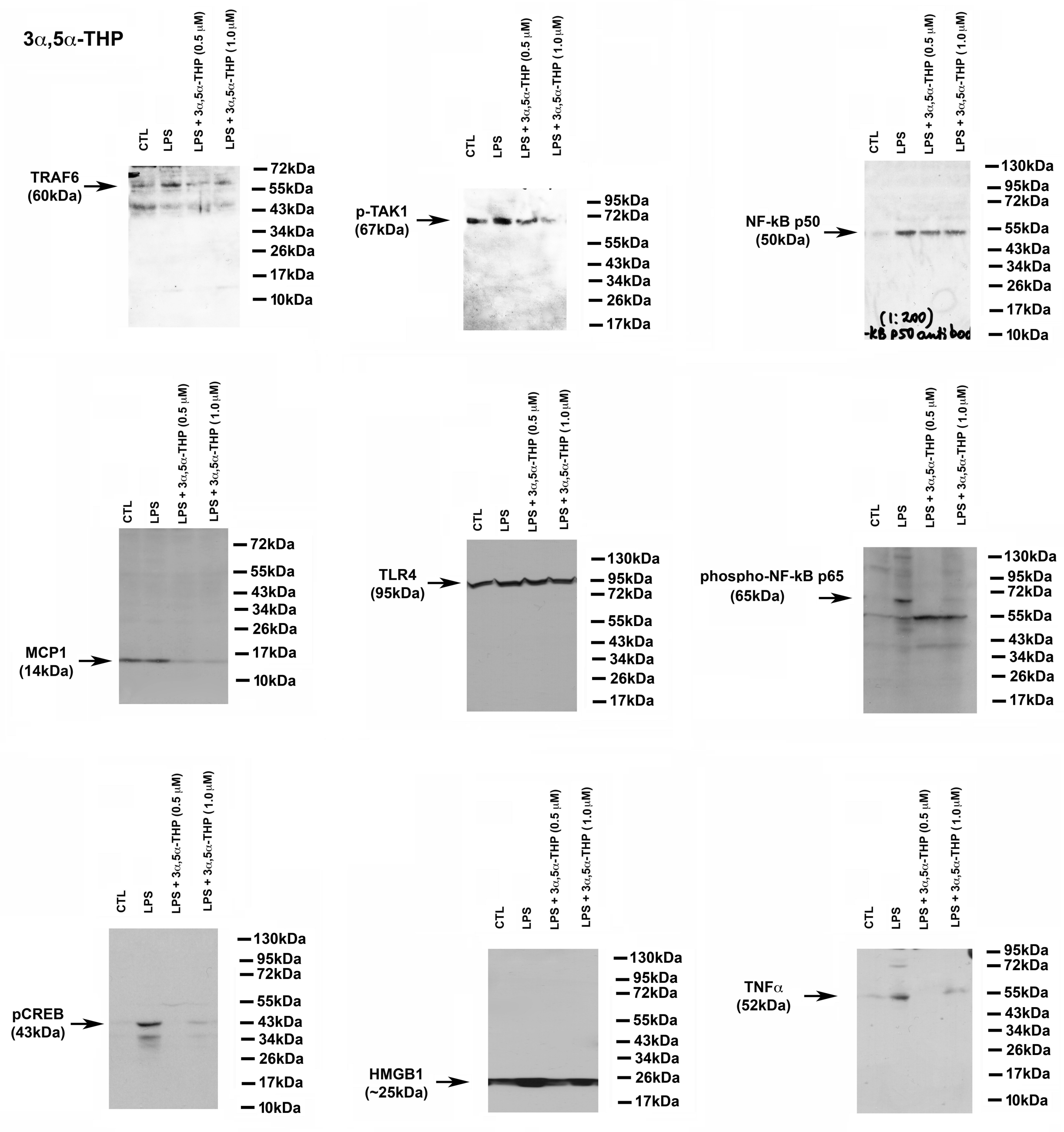

Figure S4. Original scans for the blots used in Fig 1 of the manuscript.

Manuscript Figure 2:

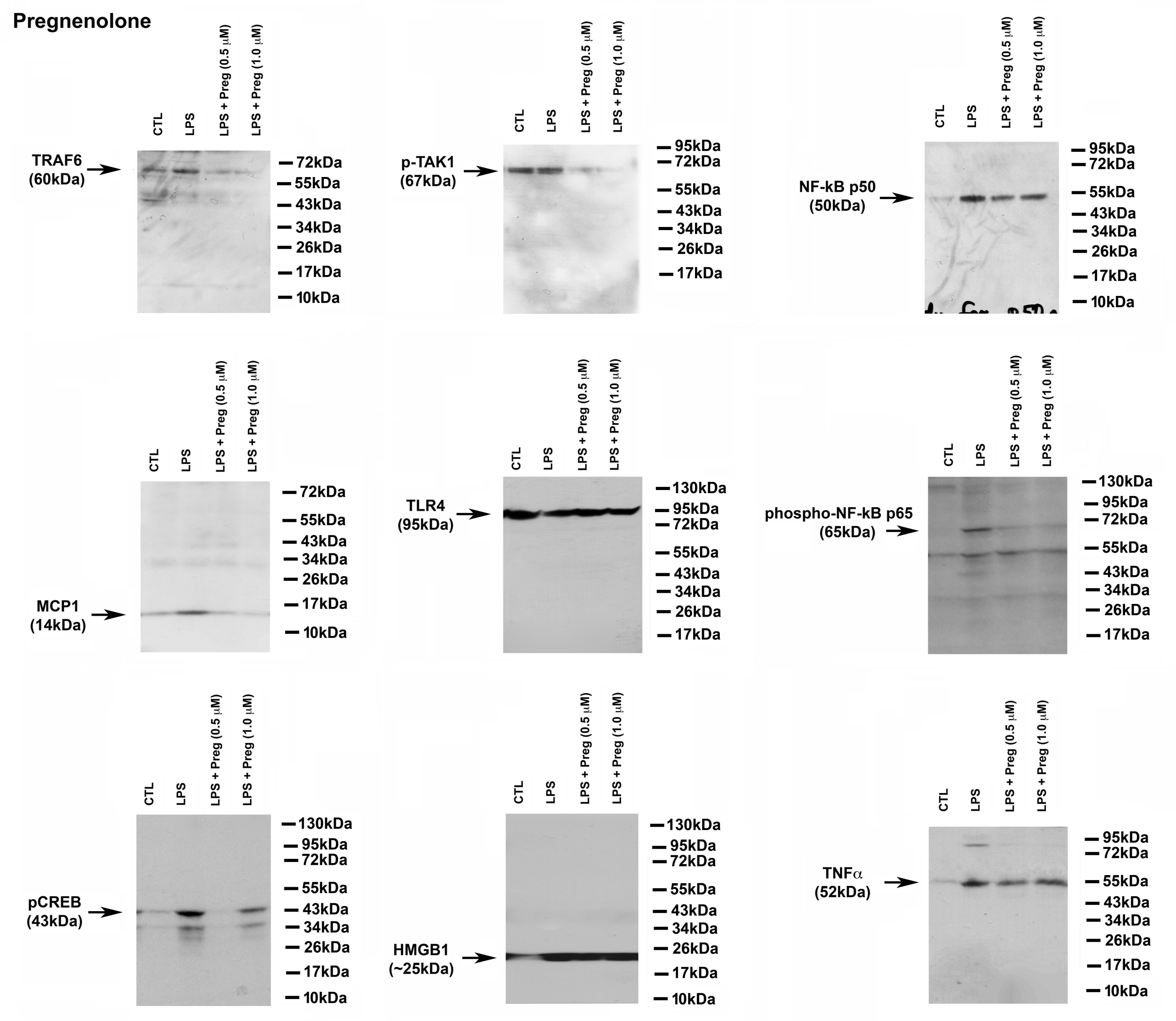

Figure S5. Original scans for the blots used in Figure 2 of the manuscript.

Manuscript Figure 3:

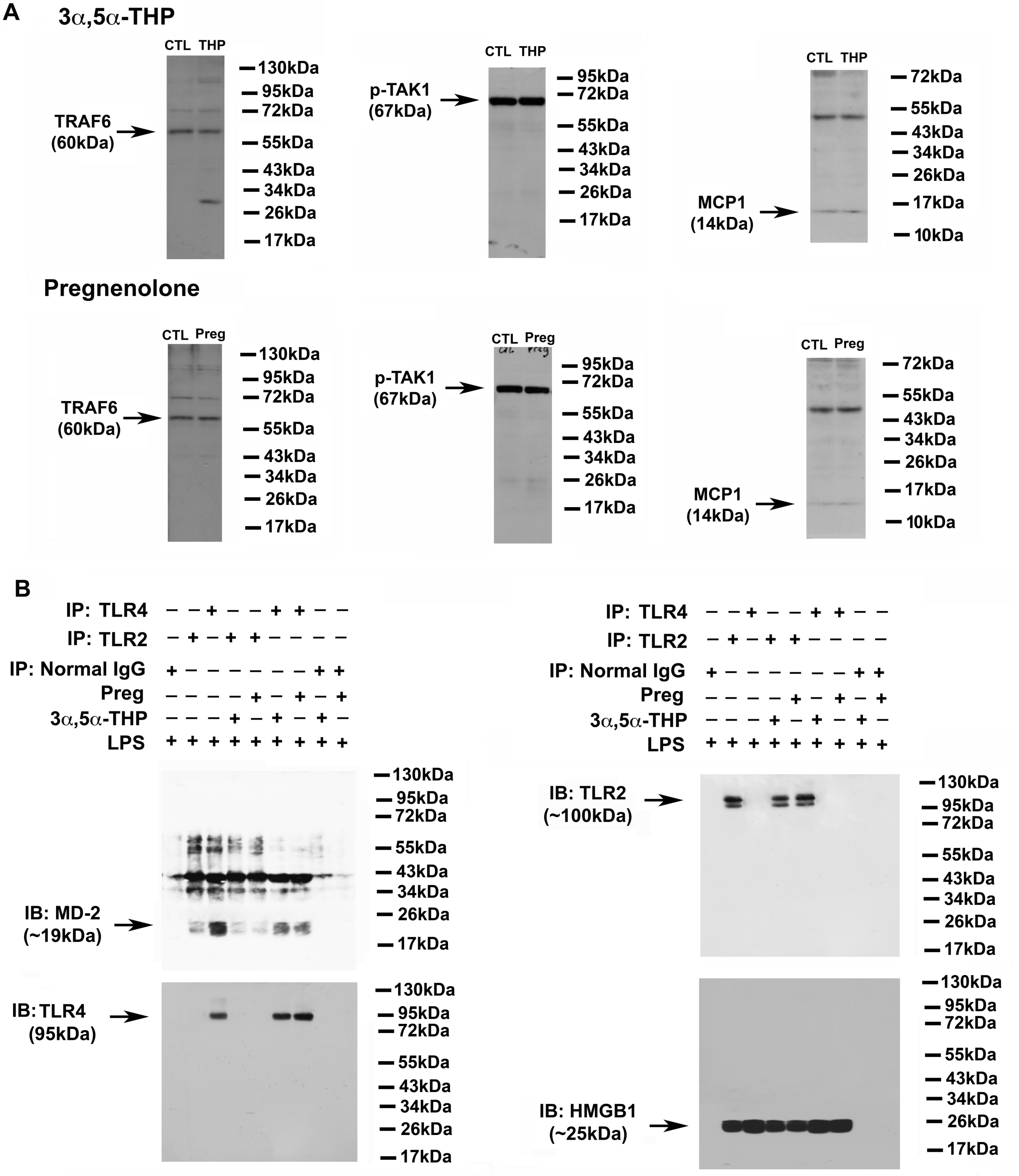

Figure S6. Original scans for the blots used in Figure 3 of the manuscript.

Manuscript Figure 4:

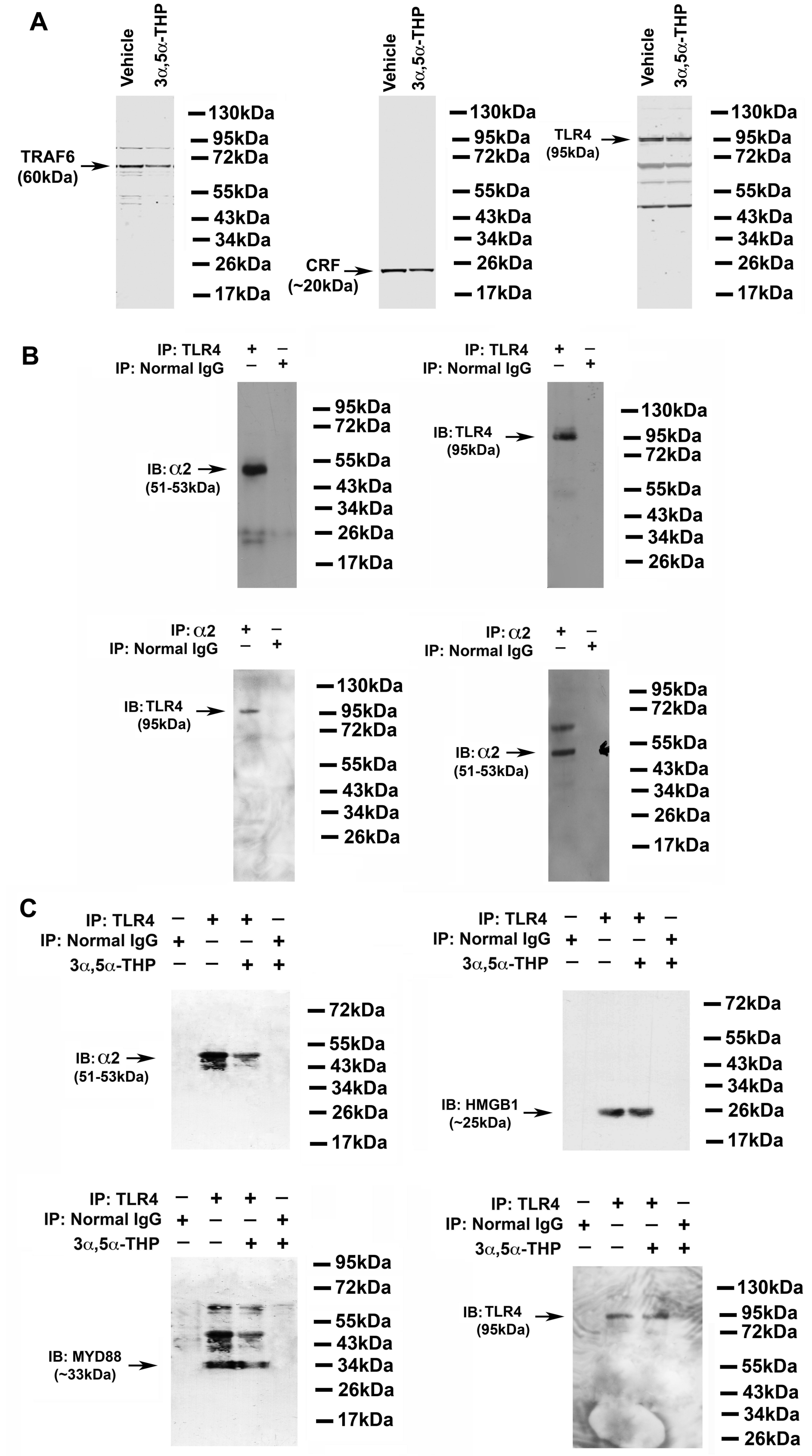

Figure S7. Original scans for the blots used in Figure 4 of the manuscript.

REFERENCES

1 Liu, J. *et al.* Binge alcohol drinking is associated with GABAA alpha2-regulated Toll-like receptor 4 (TLR4) expression in the central amygdala. *Proc Natl Acad Sci U S A* **108**, 4465-4470, doi:10.1073/pnas.1019020108 (2011).

2 June, H. L. *et al.* CRF-amplified neuronal TLR4/MCP-1 signaling regulates alcohol self-administration. *Neuropsychopharmacology* **40**, 1549-1559, doi:10.1038/npp.2015.4 (2015).

3 Aurelian, L., Warnock, K. T., Balan, I., Puche, A. & June, H. TLR4 signaling in VTA dopaminergic neurons regulates impulsivity through tyrosine hydroxylase modulation. *Transl Psychiatry* **6**, e815, doi:10.1038/tp.2016.72 (2016).

4 Balan, I., Warnock, K. T., Puche, A., Gondre-Lewis, M. C. & Aurelian, L. Innately activated TLR4 signal in the nucleus accumbens is sustained by CRF amplification loop and regulates impulsivity. *Brain Behav Immun* **69**, 139-153, doi:10.1016/j.bbi.2017.11.008 (2018).
